# Cardiovascular risk prediction in women: rethinking traditional approaches through precision medicine

**DOI:** 10.3389/fgwh.2026.1659244

**Published:** 2026-02-20

**Authors:** Zainab Atiyah Dakhil, Sama Atta Gitti, Rasha Kaddoura

**Affiliations:** 1 Al-Kindy College of Medicine, University of Baghdad, Baghdad, Iraq; 2 Department of Cardiology, Ibn Al-Bitar Specialized Center for Cardiac Surgery, Baghdad, Iraq; 3 Hamad Medical Corporation, Doha, Qatar

**Keywords:** artificial intelligence, gender, risk model, sex, women

## Abstract

Cardiovascular disease (CVD) remains the leading cause of mortality in women. Estimating cardiovascular risk using prediction models is essential for guiding preventive strategies. Despite progress, conventional risk models still omit critical women-specific factors, limiting their accuracy. Precision medicine, supported by artificial intelligence, provides a framework to integrate these overlooked determinants. This approach may help close existing gaps in cardiovascular risk prediction. Sex-specific biomarkers that contribute to overall cardiovascular risk can be incorporated into risk assessment tools to improve prevention strategies, early detection, and personalized intervention. The integration of imaging-derived variables enhances diagnosis accuracy. Moreover, pharmacokinetic modeling may help optimize therapy and reduce adverse events. Future research should focus on refining risk prediction algorithms that incorporate women-specific cardiovascular risk. Herein, we explore how addressing the burden of CVD in women through precision medicine requires a tailored approach that considers sex-specific risk factors, hormonal influences, biomarkers, and imaging modalities. This review provides a descriptive synthesis of current evidence and highlights existing knowledge gaps and future directions in precision medicine for cardiovascular risk prediction in women.

## Introduction

Cardiovascular disease (CVD) is the leading cause of death in women and accounted for 35% of all women deaths worldwide in 2019 (1). While the global prevalence of CVD in women, adjusted for age and population, has declined, the opposite trend is observed in socioeconomically disadvantaged regions (2). Cardiovascular outcomes are shaped by regional socioeconomic, environmental, and community factors, along with healthcare system and individualized factors (1, 2). Interestingly, women have lower healthcare expenditures than men in 2020 and anticipated to have lower expenditure in 2050, yet the rate of increase in costs is higher for women (224%) compared to men (173%) (3).

The care women receive for CVD, however, is often suboptimal due to various factors. These include delayed diagnoses, insufficient treatment, and gaps in knowledge, particularly concerning conditions that disproportionately affect women. For instance, spontaneous coronary artery dissection (SCAD), which is more common in women, is frequently underdiagnosed due to low clinical suspicion, especially in young, healthy women. Misinterpretation of angiographic findings also contributes to missed diagnoses (4). Furthermore, long-standing exclusion of women from clinical trials has hindered the development of sex-specific treatment protocols and guidelines, contributing to the lack of tailored healthcare for women. While this issue has improved over recent years, women remain underrepresented in clinical research, which continues to affect the quality of care they receive (1).

In clinical practice, accurately estimating and communicating cardiovascular risk is crucial for guiding preventive interventions. Current risk assessment tools, which are derived from large-scale epidemiological studies, typically include factors such as age, sex, ethnicity, cholesterol levels, blood pressure, diabetes history, and smoking status (5). However, these tools do not always account for the unique risk factors that affect women, such as hormonal influences or sex-specific biomarkers. The exclusion of women from clinical trials has exacerbated these gaps, leaving a lack of evidence on how CVD manifests differently in women and how best to treat them.

The emerging field of precision medicine offers promise in addressing these disparities. By tailoring healthcare to an individual's genetic, environmental, and lifestyle factors, precision medicine aims to improve disease prevention and treatment. With advancements in omics technologies (genomics, transcriptomics, proteomics, and metabolomics) and artificial intelligence (AI), precision medicine is poised to reduce sex disparities in cardiovascular care. This review explores how precision medicine can be leveraged to enhance cardiovascular care for women, focusing on the integration of sex-specific risk factors, hormonal influences, biomarkers, and advanced imaging techniques (6, 7).

## Search strategy

This article is a narrative review that provides a descriptive summary of the literature and offers perspectives on the existing knowledge gaps and opportunities for future refinement in cardiovascular risk prediction for women. A targeted literature search was conducted in PubMed using combinations of the following keywords: “*cardiovascular disease,” “risk prediction,” “women,” “sex differences,” “precision medicine,” “risk model,” “biomarkers,” “omics,”* “*imaging”* and “*artificial intelligence”.* Boolean operators (“AND”, “OR”) were used to capture relevant permutations of these terms.

The search was limited to English-language publications. Reference lists of key articles and recent reviews were screened to identify additional sources. Studies were included if they addressed cardiovascular risk assessment, precision medicine applications, or sex-specific predictors of cardiovascular outcomes.

Because this is a narrative rather than a systematic review, other databases (e.g., Embase, Cochrane Library, Scopus) were not included, and no formal inclusion or exclusion criteria were applied. The search did not involve a librarian. Instead, emphasis was placed on seminal studies, authoritative reviews, and recent advances published in peer-reviewed journals that illustrate emerging trends and unresolved challenges in the field. Findings were synthesized narratively with attention to discrimination, reclassification, and clinical applicability.

## Sex disparities in cardiovascular risk factors and diseases

**Figure 1:**
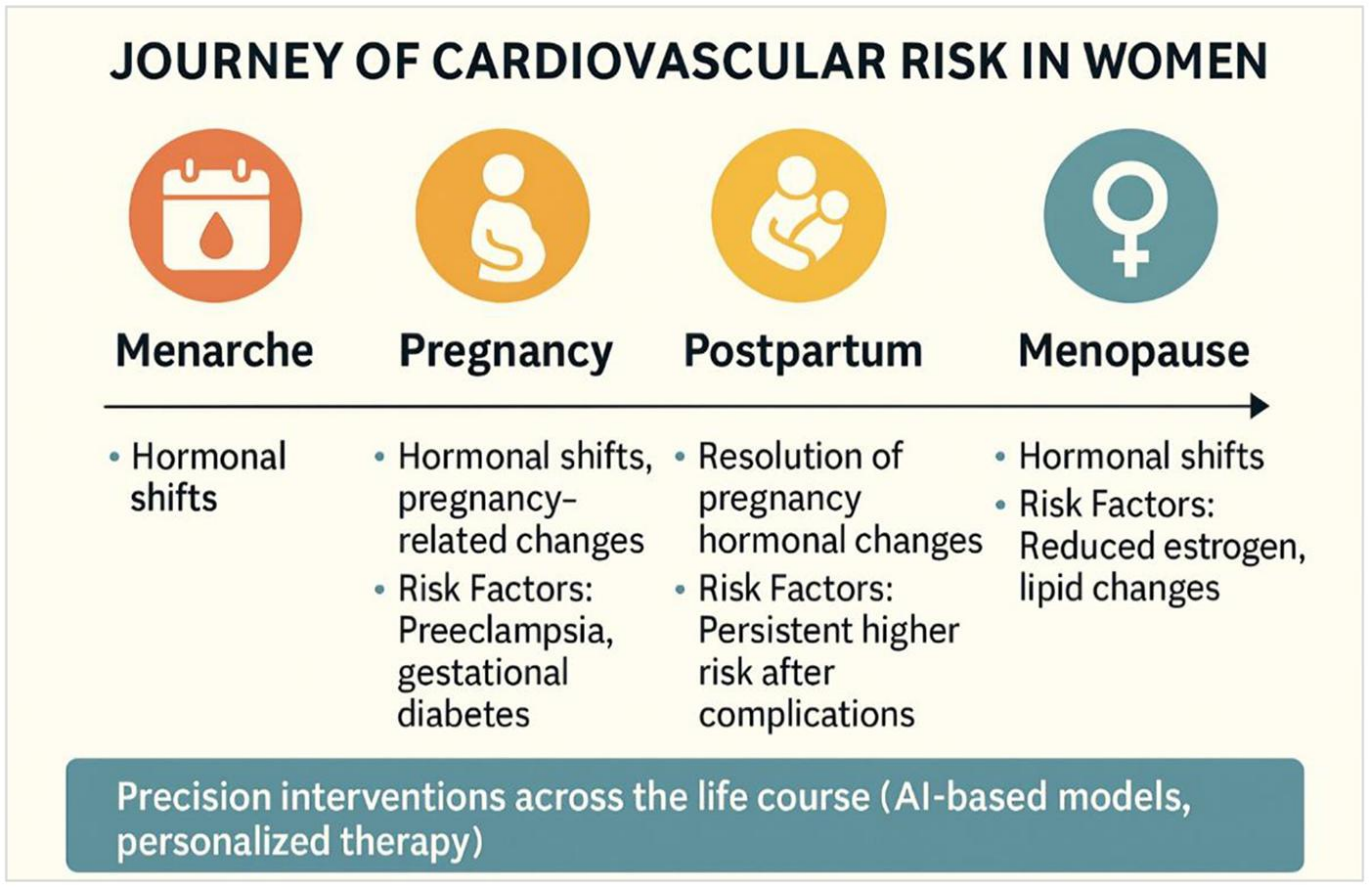
Cardiovascular risk in women across life cycle.

Women differ from men across the entire spectrum of CVD, encompassing prevalence rates, influence of risk factors, diagnostic test outcomes and their interpretations, as well as responses to medications and the incidence of adverse drug reactions (1, 8–10). In individuals over 65 years, low HDL-C is a stronger predictor of cardiovascular mortality in women than in men (11). Autoimmune diseases that are more prevalent in women such as systemic lupus erythematosus and rheumatoid arthritis, increase CVD risk due to chronic inflammation and treatment-related complications (12). Breast cancer survivors are also at augmented cardiovascular risk because of potential chemotherapy-induced cardiotoxicity (13). Women-specific conditions including pregnancy, polycystic ovary syndrome (PCOS), and menopause, are linked to cardiovascular events (Figure 1). PCOS increases CVD risk through insulin resistance, metabolic syndrome, and diabetes, whereas menopause exacerbates traditional CVD risk factors due to the associated estrogen withdrawal (14). This hormonal shift leads to adverse metabolic changes, including increased visceral fat, impaired glucose tolerance, dyslipidemia, hypertension, endothelial dysfunction, and vascular inflammation (15).

Smoking is a major cardiovascular risk factor in women, shaped by both sex-specific biological susceptibility and the social and behavioral determinants.

Despite the growing recognition of sex-specific cardiovascular risks, many widely used risk prediction models still do not fully integrate all these factors, potentially limiting their accuracy in assessing risk among women. Recognizing and integrating these factors into risk assessment is crucial for improving CVD prevention and management in women. Overall, evaluating cardiovascular symptoms in women remains challenging due to their different presentation from men. For example, chest pain in women is less predictive of obstructive coronary artery disease (CAD) than in men (16). This disparity was first highlighted nearly 40 years ago when women who underwent coronary angiography for atypical ischemic symptoms had lower rates of obstructive CAD than men (17). Additionally, women with established CVD frequently report nonspecific symptoms, such as fatigue and sleep disturbances, which may obscure diagnosis and lead to misinterpretation (18). A particularly harmful misconception in cardiovascular practice is the tendency to attribute women's symptoms to psychological causes, such as anxiety or panic attacks. Women are twice as likely as men to be discharged from the emergency room without appropriate cardiac evaluation, despite presenting with acute coronary syndrome (ACS) symptoms (18). This diagnostic oversight underscores the urgent need for greater awareness and accuracy in assessing CVD in women.

## Smoking and cardiovascular risk in women

Observational data demonstrates that smoking initiation, smoking continuation, and higher smoking intensity are associated with higher cardiovascular disease risk in females and males, with directionally similar sex-specific excess risks for coronary heart disease (19). While a large systematic review and meta-analysis involving nearly 4 million participants found that, compared with nonsmokers, women who smoke had around 25% greater relative risk of coronary heart disease than male smokers after adjusting for other cardiovascular risk factors, although the mechanisms underlying the sex differences remain unclear (20). Importantly, recent population-based evidence indicates that these risks may be amplified by emerging gender-related trends in tobacco use. In a large 10-year population study from England, smoking prevalence increased among women of reproductive age (18–45 years), particularly in younger and more socioeconomically advantaged groups (21). These findings highlight how changing social norms, targeted marketing, and evolving nicotine use behaviors may be contributing to a renewed burden of smoking-related cardiovascular risk in younger women, underscoring the need for cardiovascular risk prediction and prevention strategies that explicitly integrate both sex-specific biological effects and gender-related behavioral dynamics.

## Cardiovascular risk in postmenopausal women

Women who experience early or surgical menopause are at risk of an earlier loss of cardiovascular protection, making their cardiovascular risk profile similar to that of men at the same age group (22). Postmenopausal women may benefit from an active earlier screening, including coronary calcium scoring and biomarkers testing (23), to detect subclinical atherosclerosis and guide timely preventive strategies. Proactive care during this transitional period may improve long-term cardiovascular outcomes and enhance overall health and quality of life. Postmenopausal women possess a distinct range of non-hormonal cardiovascular risk factors that are either exclusive to or prevalent in females. A history of adverse pregnancy outcomes (e.g., pre-eclampsia, gestational hypertension, and gestational diabetes) is associated with an increased risk of future CVD (1). Autoimmune disorders such as systemic lupus erythematosus, rheumatoid arthritis, and Sjögren's syndrome are more common in women and are evidently linked to chronic inflammation, endothelial dysfunction, and early atherosclerosis (24). Women with a history of breast cancer who undergo treatments with anthracyclines, trastuzumab, and chest radiotherapy may face an increased cardiovascular risk profile, making CVD the top cause of mortality among older breast cancer survivors (25). Additionally, women who have a background of PCOS continue to face enduring cardiometabolic risks (e.g., insulin resistance, dyslipidemia, and hypertension) during menopause, even when reproductive signs of PCOS diminish (26). Disruptions in sleep, especially insomnia and obstructive sleep apnea (27, 28), also rise following menopause because of airway changes and modified fat distribution, hence, becoming new sex-specific factors contributing to hypertension and arrhythmias (29). Sarcopenia due to complex mechanisms that lead to imbalance between anabolic and catabolic muscle homeostasis with or without neuronal degeneration, is associated with faster progression of CVD (30). Vasomotor symptoms associated with menopause (e.g., hot flashes and night sweats) have been associated with increased sympathetic activity, insulin resistance, and endothelial dysfunction thus, acting as clinically important sex-specific markers of cardiovascular risk (31, 32).

## Cardiovascular risk in pregnancy

In pregnancy, CVD presents a complex clinical and research challenge due to specific hemodynamic and physiological adaptations, heterogeneous risk factors, and limited high-quality data. A major barrier to advancing research is the historical exclusion of pregnant individuals from clinical trials due to ethical concerns and liability risks, resulting in huge knowledge gaps regarding diagnosis, risk stratification, and treatment (33), necessitating more sex-focused registries and trials to bridge these gaps (34, 35). Up to 4% of pregnancies can have cardiovascular complications, with CVD constituting 26.5% of pregnancy-related deaths (33). Cardiac risk during pregnancy should be assessed on an individualized basis. For example, maternal age above 30 years, pre-eclampsia, coronary artery dissection, gestational diabetes, blood transfusion and peripartum infection, can increase the risk of ACS during pregnancy (36). Moreover, the underlying pathology for ACS in pregnancy can differ. As an example, coronary artery dissection is the most common cause of pregnancy associated ACS (37), which calls for a more individualised risk stratification and management approach in pregnant women. Not only is the etiology of CVD in pregnancy different, but diagnosis can also be challenging, for instance, pregnant patients with ACS often present with atypical symptoms (e.g., vomiting, reflux, diaphoresis) that may mimic the physiological changes of pregnancy, pregnancy-related conditions (e.g., pre-eclampsia), or both (38). In addition, pregnant patients may present with more hemodynamic compromise, arrhythmia, or cardiogenic shock (38).

## Contemporary cardiovascular risk scores

### Prediction models for CVD risk in the general population

Early identification of individuals at high CVD risk is paramount in preventing and reducing burden of CVD (39, 40). Various strategies have been implemented with risk scores being important tools that guide identification of cardiovascular risk and initiation of therapy (39). Damen et al. in their systematic review of 212 articles described 363 prediction models for CVD risk with 36.4% of the models that were validated (41). Smoking and age were the most frequent predictors in 89.5% and 88.4% of the models, respectively, and 68.9% of the models were sex-specific (41). The most popular cardiovascular risk scores in the general population include the Framingham Risk Score (FRS) that predicts 10-year cardiovascular risk (42), Systematic COronary Risk Evaluation-2 (SCORE2) (43), SCORE2-OP (44), Pooled Cohort Equations (45), and QRISK scores (e.g., versions QRISK3 and QRISK4) (46, 47). In addition, risk prediction scores in specified populations such as those with diabetes mellitus (48) and prior CVD (49) have been developed. Damen et al. found a remarkable heterogeneity between the models in terms of predictors and outcomes definitions (41). Furthermore, the practicality of most models remains uncertain due to the methodological limitations, incomplete presentation, and lack of adequate studies on models’ impact or external validation (41).

### Prediction models for CVD risk in women

The risk prediction of CVD morbidity and mortality differs between men and women which may reflect differences in the modifiable risk factors distribution, pathophysiological mechanisms, clinical presentation, disease course, and clinical outcomes severity (39, 40, 50). Thus, sex differences should be considered in CVD risk scores (39). Several risk prediction models have been developed in women but the differences in CVD risk assessment between men and women are not well recognized (40). Earlier, a version of the Reynolds risk score that assessed cardiovascular risk in healthy women of 45 years or older predicted 10-year CVD risk by using age, smoking, systolic blood pressure, total cholesterol, HDL-C, high-sensitivity (hs) C-reactive protein (CRP), family history of premature myocardial infarction, and glycated hemoglobin in women with diabetes. This score improved accuracy that reclassified up to 50% of women at intermediate risk into lower- or higher-risk categories (51). The addition of coronary artery calcification to established risk prediction models may help identifying asymptomatic women (52) or low-risk women who are at higher risk according to risk stratification approaches (53). Zhou et al. constructed the NEW-STROKE model by adding the use of HRT among six other factors (e.g., ethnicity, exercise, height, etc.) to the established Framingham Stroke Risk Score for postmenopausal women (54). They reported an improved stroke risk prediction in women but the impact of hormone use alone was not reported (54). Sedlak et al. reported that the commonly used CVD risk (55) prediction scores cannot precisely predict CVD rates in women presenting with ischemia who do not have obstructive CAD. Several biomarkers have been evaluated to improve CVD risk prediction (56). Wang et al. measured 10 biomarkers [e.g., CRP, B-type natriuretic peptide, N-terminal pro-atrial natriuretic peptide (NT-proBNP), aldosterone, etc.] with only moderate addition to standard risk factors (57). Everett et al. showed that NT-proBNP modestly improved prediction of CVD risk in women (58). Wiviott et al. suggested that multi-marker approach identified a larger proportion of high-risk women in the setting of unstable/non-ST segment elevation myocardial infarction (59). Ridker et al. used a single combined measure of biomarkers [i.e., hs-CRP, low-density lipoprotein cholesterol (LDL-C), and lipoprotein(a)] levels in healthy women which predicted the incidence of cardiovascular events over a 30-year follow-up period (60).

Goh et al. reviewed six risk prediction models in women and reported that important risk factors were generally not included in the models such as physical activity, alcohol consumption, obesity, chronic kidney disease, antihypertensive drugs use, and coronary artery calcium (CAC) (50). Baart et al. conducted a systematic review of 285 prediction models for women in the general population. Of them, 160 (56.1%) models were female-specific (i.e., for use in women only) and 125 (43.9%) models were sex-predictor (i.e., included sex as a predictor). The median number of predictors for models was 6–8 (e.g., age, smoking, diabetes mellitus, systolic blood pressure, hypertension, diastolic blood pressure, total cholesterol, LDL-C, and HDL-C) (40, 61). Baart et al. (40) found that only two models included female-specific predictors; menopause in one model (61) and key reproductive-related factors in another (62), while three models added female-specific risk factors to an existing model (54, 62, 63). Overall, it is uncertain that incorporating female-specific predictors can improve risk prediction scores (40). Recently, van Os et al. developed prediction models in men and women aged from 30 to 49 years for the first-ever cardiovascular event (64). They concluded that sex-specific prediction models had moderate discriminatory performance and identified nontraditional cardiovascular predictors, that only modestly increase prediction models’ performance (64). Amiri et al. in their recent study assessed the validity of FRS in women with PCOS and demonstrated a significant increase in risk of CVD in women with PCOS. The FRS predicted an increase of 38% in CVD risk for every one-unit increase in FRS (65).

### Prediction models for CVD risk in pregnant women

The development of adverse pregnancy outcomes or complications puts women at an increased risk of CVD (e.g., stroke and myocardial infarction), with an immediate risk to fetal and maternal health (66–68). In a large study (*n* = 2,134,239), pregnancy complications (i.e., preeclampsia or eclampsia, small for gestational age, gestational or hypertension, stillbirth, and preterm birth) were associated with all-cause death, cardiovascular death, and hospitalizations for CVD (69). The reproductive history of a woman is an important factor to be evaluated and has been considered in the relevant international guidelines (70–73). However, CVD risk stratification and assessment in women is complex and may underestimate the risk, namely in young women (66). Moe et al. reported that three popular scoring systems were inadequate in assessing CVD risk one-year postpartum (74).

Risk prediction models for CVD in pregnancy are needed to predict the related cardiovascular complications. Contemporary models that are used in practice incorporate clinical, demographic, and imaging parameters to improve risk assessment and guide management plan in this population (75, 76). The most well-established models include the CARPREG I and II, ZAHARA I and II, the World Health Organization (WHO) Maternal Risk Classification, and the modified WHO (mWHO) Classification System (75, 77).

Several studies have investigated whether incorporating the history of pregnancy complications in the scoring systems would improve CVD risk prediction. Stuart et al. added hypertensive disorders of pregnancy to an established CVD risk prediction score and did not find improvement in discrimination or reclassification (78). Timpka et al. did not show meaningful improvement in 10-year CVD risk prediction when adding hypertensive disorders of pregnancy or delivering low birth weight offspring to an established risk score in women aged 50 years or older (79). Parikh et al. incorporated pregnancy-related (i.e., pregnancy status, number of live births, age at menarche, menstrual irregularity, infertility ≥ 1 year, infertility cause, age at first birth, stillbirths, miscarriages) and breastfeeding predictors in an established risk model. They found that incorporating such key reproductive factors only very modestly improved model discrimination but not reclassification in association with coronary heart disease (CHD) (62). Markovitz et al. also added history of pregnancy complications (i.e., small for gestational age, pre-eclampsia, gestational hypertension, or preterm delivery) to an established risk model and only detected small improvement in CVD risk prediction driven by pre-eclampsia. Pre-eclampsia alone independently predicted CVD (80). When van der Meer et al. incorporated the female-specific predictors (i.e., hormone use, age at menarche, gestational hypertension and diabetes, number of children, miscarriages and still births, and menopausal status and age) into two Dutch population-based studies (PROSPECT and MORGEN) (81) found no added value in terms of improving 10-year CVD risk prediction in women (74). Tanz et al. concluded that including preterm delivery and parity into CVD risk prediction scores resulted in small improvement in risk prediction which may be most useful in young women (82). Saei et al. found that adding history of adverse pregnancy outcomes (i.e., history of ectopic pregnancy, placenta previa, placenta abruption, preterm delivery, abortion, stillbirth, preeclampsia, gestational hypertension, and diabetes) to FRS improved the prediction of CVD (83). Interestingly, Doust et al. in their recent study included female-specific risk factors (e.g., early menarche, irregular menstruation, endometriosis, miscarriage, stillbirth, infertility, pre-eclampsia, gestational diabetes, and early menopause) in three popular CVD risk scores. The investigators showed that female-specific factors can be early indicators of CVD risk (84).

Overall, the studies showed limited incremental benefit of CVD risk prediction when adding reproductive- or pregnancy-related factors to established risk prediction models (39, 67). Such lack of meaningful improvements in predictive performance can be due to many factors. Firstly, these complications often reflect underlying cardiometabolic disturbances (such as hypertension, insulin resistance, and endothelial dysfunction) that are already captured by traditional risk factors included in existing models. Secondly, the cardiovascular consequences of pregnancy complications may manifest over a longer time horizon than the 10-year period typically used in risk prediction, limiting their short-term prognostic value. Thirdly, these complications lack specificity; not all women with adverse pregnancy outcomes will develop CVD, and many who do have no such history, reducing their utility as discriminative predictors. Fourthly, the underrepresentation or inconsistent documentation of reproductive history in many population-based cohorts used for model development may attenuate their apparent impact. Finally, conventional risk models are already statistically robust and saturated with high-performing predictors, meaning that even clinically relevant variables may contribute little incremental value in terms of overall risk prediction accuracy.

In women with pre-existing cardiac disease, pregnancy increases cardiovascular complications (85). Currently, there are several risk scores (86–89) and a lesion-specific classification system (i.e., mWHO classification) (90). The mWHO classification is a useful tool for women with established cardiac disease to predict cardiac events during pregnancy (90). Hameed et al. integrated an established CVD risk assessment tool into electronic health record and proposed an innovative approach for a universal assessment of CVD risk during pregnancy and postpartum. The investigators aimed at early identification of high-risk patients and follow-up to improve maternal clinical outcomes (91). Pande et al. established and validated the utility of two risk stratification tools to predict adverse cardiac outcomes in pregnant women with valvular heart disease. The tools demonstrated good clinical utility and discriminative ability (92). Wambua et al. published their study protocol in which they are developing a CVD risk prediction model for CVD postpartum that incorporates pregnancy-related risk factors that have not been considered in the current risk prediction models (93).

## Limitations of current cardiovascular risk prediction models

Cardiovascular risk assessment is usually done using the forementioned traditional risk scores (e.g., FRS, SCORE, QRISK, etc.) that were validated in their studied populations and each one has its advantages and limitations. For example, risk scores do not usually incorporate family history which is a key cardiovascular risk factor (56, 94). Age bias often exists with a general tendency to underestimate the cardiovascular risk in younger population and overestimate it in their older counterparts (94). For instance, the SCORE score that predicts the 10-year risk of cardiovascular mortality was developed from 12 European cohort studies (*n* = 205,178) with 7,934 cardiovascular deaths. The SCORE score considers the following parameters: age (range of 40–65 years), sex, systolic blood pressure, total cholesterol, and smoking status. The age range is 40–65 years, and patients with established CVD or diabetes were excluded (95, 96). An important limitation of the SCORE score is that it is not applicable in patients above 65 years of age. More specifically, it overestimated cardiovascular mortality risk in subjects aged 65–69 years and in normotensive subjects, whereas it underestimated cardiovascular mortality risk in hypertensive patients and in subjects aged 70–79 years (41, 97). Emerging use of novel biomarkers (e.g., CRP, coronary calcium, and microalbuminuria) have been identified, but their overall contribution to risk models remains limited (41). Thus, precision medicine, by leveraging novel biomarkers and artificial intelligence, represents an emerging and transformative strategy for enhancing CVD risk stratification and patient outcomes.

## Omics in precision cardiology

With molecular, physiological, and environmental information being available, cardiology is moving beyond the traditional “one-size-fits-all” paradigm towards the individual- and population-specific insights to adopt new genetic, molecular, metabolic, and proteomic tools (6). The integration of omics technologies (i.e., genomics, transcriptomics, proteomics, metabolomics, and epigenomics) has transformed cardiovascular medicine by enabling personalized risk stratification and therapeutic targeting (98). Traditional cardiovascular risk prediction models, such as the FRS or SCORE score, rely mainly on demographic and clinical parameters, which often lack accuracy across diverse populations (99). In contrast, the genome-wide association studies have identified more than 160 genetic loci associated with CAD enhancing our understanding of genetic susceptibility (100). This has led to the development of polygenic risk scores, which integrate the cumulative effect of multiple genetic variants to quantify individual cardiovascular risk (99). High polygenic risk score levels have been shown to triple the risk of CAD, even among individuals with no traditional risk factors, making it a valuable tool for an early intervention (99). Transcriptomic profiling contributes further by capturing gene expression changes linked to inflammation, fibrosis, and vascular remodeling, offering prognostic information beyond the static genotypes (101). Proteomic analyses identify circulating proteins such as NT-proBNP and galectin-3, which are now clinically used to assess heart failure severity and guide treatment decisions (101). Metabolomics has revealed specific metabolic biomarkers, including elevated levels of trimethylamine N-oxide, which are associated with increased risk of atherosclerosis and adverse cardiovascular events (102). Epigenomic studies have shown that DNA methylation and histone modifications influence cardiovascular phenotypes, often reflecting lifestyle and environmental exposures (103). Regulation of genes by transcription factors, alongside epigenetic mechanisms, is fundamental to the pathophysiology and progression of atherosclerosis with an interplay between different regulatory systems (104). These systems can be modulated through several distinct mechanisms such as epigenetic modifications and epigenetic changes as well as transcription factors that can drive chromatin remodeling by recruiting and interacting with various epigenetic modifiers (104). By combining multi-omics data with clinical variables and machine learning tools, personalized and continuously adaptive risk prediction models can be developed, moving cardiovascular medicine toward a truly precision-based paradigm.

## Phenotyping in cardiovascular care

With the advent of precision medicine, advanced phenotyping of patients and population has led to the recognition of the inherent heterogeneity among individuals who are at risk for or with established CVD (7). Leopold et al. identified two distinct phenotypes in women using hierarchical clustering and phenomap analysis. Women in the first cluster were characterized by higher cardiovascular risk, and were older, had a lower affluence index, and exhibited higher weight, body mass index, blood pressure, glucose, and cholesterol levels, consuming less heart-friendly diet, leading to a higher incidence of cardiovascular events compared to those in the second cluster (105). Compared to men, women in the higher-risk cluster shared similar age but had a higher body mass index and were more likely to be smokers, with no difference in blood pressure or blood glucose, which suggested that females have lower health score compared to their male counterparts (6). A study by Akintunde et al. found that physiologically individualized therapy based on renin/aldosterone phenotyping significantly improved blood pressure control (106). The study concluded that black patients of an African origin often have a genetically determined predisposition to salt and water retention and suppressed plasma renin activity (Liddle phenotype) for which amiloride may be the suggested therapy (106). In primary hyperaldosteronism phenotype, aldosterone antagonists are considered the best medical therapy while for renal phenotype the main treatment will be renin-angiotensin-aldosterone system blockers (106).

## Precision medicine for predicting CVD in women

Despite that there are sex-based physiological differences in left ventricular mass, cardiac output, and heart rate which may influence disease phenotypes, these differences are not only driven by hormonal influences. They are also driven by inherent genetic factors such as distinct sex-chromosome compositions and the expression of escape genes that evade X-chromosome inactivation (107). In addition, there are epigenetic processes in mediating sex differences in the cardiac pathology (107). These can be revealed by the detailed analyses of DNA methylation, histone modifications, and chromatin organization. However, global epigenetic landscapes appear similar between the hearts of healthy men and women (107). Such localized differences may contribute to the sex-specific susceptibility and progression of various cardiac diseases, such as dilated cardiomyopathy and heart failure (107, 108). In a study included 27,939 initially healthy women who were followed for 30 years for the primary outcome (i.e., first major adverse cardiovascular event, a composite of myocardial infarction, coronary revascularization, stroke, or death from cardiovascular causes), it was found that biomarkers such as hs-CRP, LDL-C, and lipoprotein(a) may be useful predictors of cardiovascular risk. Hazard ratios were 1.70 [95% confidence interval (CI): 1.52–1.90] for hs-CRP, 1.36 (95% CI: 1.23–1.52) for LDL-C, and 1.33 (95% CI: 1.21–1.47) for lipoprotein(a). Each biomarker showed independent contributions to the overall cardiovascular risk (60). Biomarkers can be implemented as predictors of CVD in women with sex-specific risk factors. In women with a history of pre-eclampsia and HELLP (Hemolysis, Elevated Liver enzymes, and Low Platelets) syndrome, certain biomarkers were found to be associated with higher risk of CVD in women. For example, biomarkers such as soluble fms-like tyrosine kinase-1, placental growth factor, interleukin (IL)-6, IL-6/IL-10 ratio, high-sensitivity cardiac troponin I, activin A, soluble human leukocyte antigen G, pregnancy-associated plasma protein A and norepinephrine were potential predictors of hypertension and CVD in this population (109).

## Precision medicine and artificial intelligence

Artificial intelligence has emerged as a promising tool to improve the accuracy and efficiency of precision medicine in the cardiovascular field by analyzing large datasets. Thus, AI can improve diagnosis [i.e., data interpretation of electrocardiogram (ECG), echocardiography, cardiac computed tomography angiography (CCTA), and cardiac magnetic resonance (CMR)], risk prediction, and treatment strategies (110). However, the use of AI in precision medicine is faced with challenges such as the privacy and confidentiality of data, concerns over cyber security, the need for high-quality data sets, regulatory aspects, and ethical consideration of an AI-driven healthcare. AI-powered ECG analysis can accurately detect cardiac structural and morphological characteristics solely from ECG data which are traditionally assessed through imaging modalities such as echocardiography or CMR. AI-powered ECG could predict these parameters independently of imaging (111). One of the latest applications of the AI-driven risk prediction in women is the sex discordance score which is an emerging and potential tool that can be used in real practice for cardiovascular risk stratification in women with a performance comparable to that of the already established risk prediction models (e.g., PREVENT equation) (111, 112). While also incorporating female-specific cardiovascular risk factors, such model if validated, it can then emerge as a powerful tool for a personalized cardiovascular risk assessment in women (111). Such models can compose a major step in large-scale risk evaluations to inform public health strategies, especially in low-resource settings.

Echocardiography remains the cornerstone of cardiovascular imaging due to its wide accessibility, cost-effectiveness, and ability to assess structural and functional cardiac abnormalities. Studies have demonstrated that stress echocardiography can predict major adverse cardiovascular events in women with suspected CAD and more advanced strain imaging using speckle-tracking echocardiography has been increasingly recognized as a powerful tool for detecting subclinical myocardial dysfunction in high-risk women, including those who are undergoing chemotherapy for breast cancer (113, 114).

In two population-based cohorts, i.e., MESA (Multi Ethnic Study of Atherosclerosis) and DHS (Dallas Heart Study), that included 7042 White, Black, and Hispanic patients who underwent CAC testing, patients were followed for 10 years to detect incident ASCVD events (115). The results found increasing CHD and stroke rates across increasing CAC score which were significant across all sex and race groups (115). However, Lakoski et al. examined CAC in women classified as “low risk” and assessed their actual risk of CHD and CVD. Among the 3,601 women of a mean age of 60 years enrolled in MESA, 2,684 of them were low-risk women without diabetes mellitus. Despite being categorized as low risk, 32% of them had detectable CAC, which was strongly associated with an increased risk of adverse cardiovascular outcomes. In addition, they had a significantly higher risk of CHD (hazard ratio 6.5, 95% CI: 2.6–16.4) and CVD events (hazard ratio 5.2, 95% CI: 2.5–10.8) than those without detectable CAC. Those with advanced CAC had absolute CHD and CVD risks of 6.7% and 8.6%, respectively, at 3.75-year follow-up (53). Including CAC with other variables in cardiovascular risk prediction models can help in establishing patient-centered preventive plans (53). Other non-invasive testing such as CCTA provides accurate assessment of the coronary arteries anatomy and atherosclerotic plaque burden. CCTA can also detect ischemia with no obstructive coronary arteries which is more prevalent in women than in men (116). Other imaging modalities including Positron Emission Tomography (PET) provides high sensitivity detection of myocardial abnormalities and endothelial dysfunction as well as providing important prognostic data when compared to traditional risk factors and anatomical imaging in women especially in the detection of microvascular dysfunction (117, 118). In summary, each cardiovascular imaging modality (e.g., echocardiography, CCTA, CMR and PET) can offer unique advantages in detecting subclinical disease, assessing myocardial function, and identifying high-risk patients. The integration of imaging-derived variables into risk prediction models has the potential to enhance individualized cardiovascular risk assessment in women which can significantly improves the predictive accuracy of traditional models. Future research should focus on refining risk prediction algorithms that incorporate multimodality imaging data for a more precise assessment of female-specific cardiovascular risk (Table 1).

**Table 1:** Pros and cons of contemporary cardiovascular risk prediction models.

Contemporary cardiovascular risk prediction models						
Key features	Traditional RM FRS, SCORE Mainly consider demographics, cardiovascular risk factors and lab investigations	Biomarker-based RM QRISK2, QRISK3 Includes a broader range of risk factors and incorporates biomarkers	Genetic RM Polygenic risk score Use genetic information to assess CVD risk	AI/ML-derived RM Use machine learning algorithms to develop risk prediction models from large datasets	Imaging-based RM Use imaging techniques to assess CVD risk	Precision medicine RM Integrate multi-Omic data, AI, and imaging to tailor risk assessment and treatment
Pros	• Widely available • Easy to calculate • Validated in various populations	• Enhanced risk stratification • Incorporate socioeconomic factors	• Can identify individuals at high genetic risk • May improve risk prediction in younger individuals	• Use wide range of variables, including unstructured data (e.g., text notes) • High accuracy • Can identify complex interactions between risk factors • AI-powered ECG analysis can accurately detect cardiac structural and morphological characteristics • Incorporate female-specific risk factors	• Strong predictor of CVD events • Improve risk stratification • Provide data on cardiac anatomy and structure • Some modalities can be accessible and bed-side e.g., echocardiography	• Incorporate sex-specific risk factors, biomarkers, advanced cardiovascular imaging modalities, and AI-driven analytics • Facilitates targeted risk stratification and guide therapy by addressing the unique molecular and physiological differences in female cardiovascular health
Cons	• Limited accuracy in certain subgroups • Overestimates risk in some populations • Does not consider sex-specific risk factors • Does not usually incorporate novel biomarkers or imaging data	• More complex calculation • Requires access to electronic health records • May not be generalizable to all populations	• Limited predictive power when used alone • Require genetic testing • Performance varies across different ethnicities. • Not fully account for sex-specific genetic factors	• Requires large datasets • Risk of overfitting • Interpretability can be challenging • Regulatory and ethical considerations • Data privacy and security concerns	Some modalities requires contrast dye, involves radiation exposure, more expensive	Require further research and development to improve accessibility and address ethical considerations

RM, risk model; FRS, framingham risk score; AI, artificial intelligence; ML, machine learning; ECG, electrocardiogram; CVD, cardiovascular disease.

## Precision medicine and tailored pharmacological therapies in women

**Figure 2:**
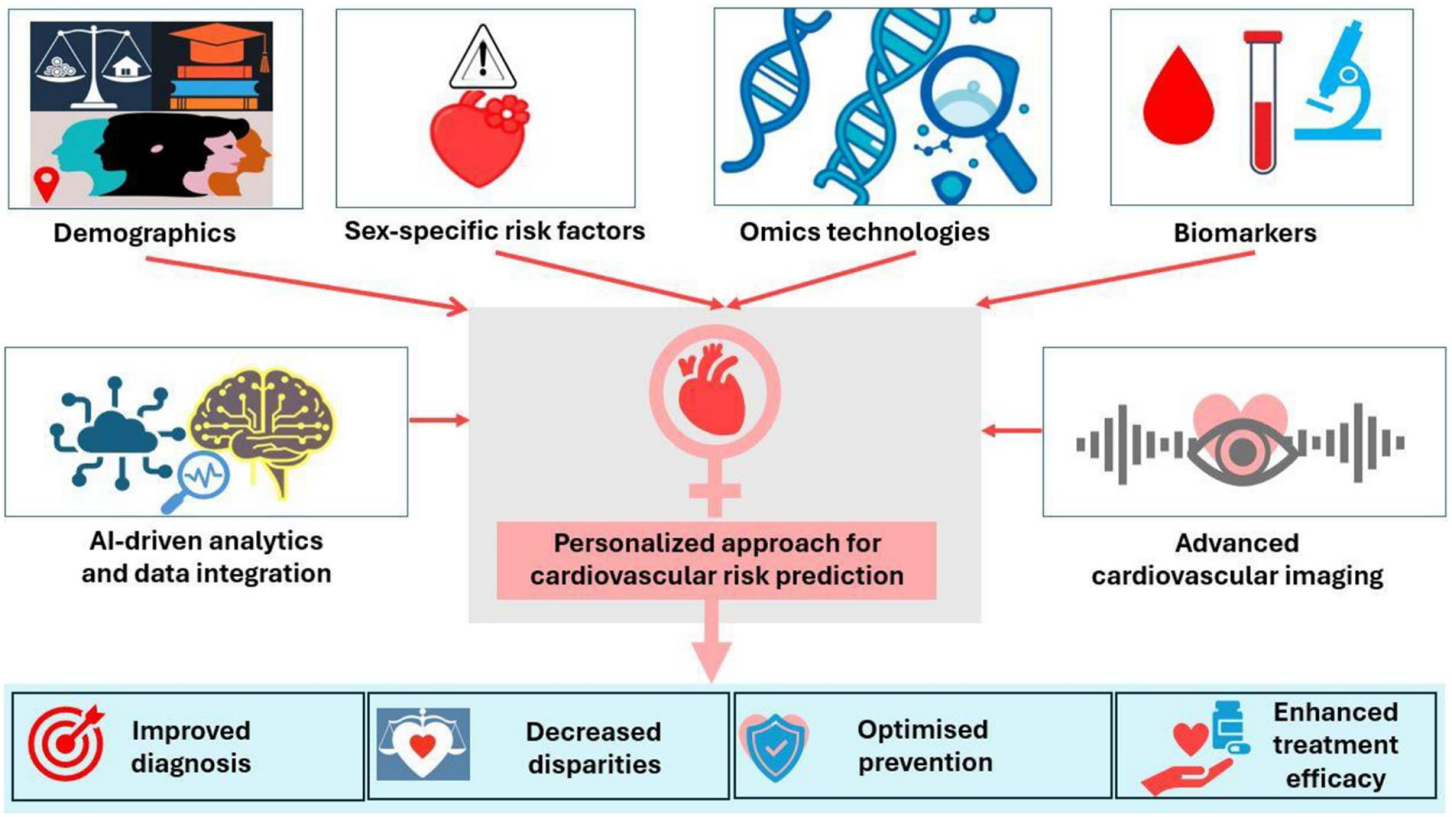
Precision medicine in cardiovascular risk prediction in women.

Pharmacogenomic studies demonstrated that genetic polymorphisms in drug-metabolizing enzymes significantly affect cardiovascular therapy in women. For example, cytochrome (CYP) 2C19 polymorphisms influence clopidogrel metabolism, hence, alternative antiplatelet strategy is required (119). Similarly, SLCO1B1 variants alter statin pharmacokinetics and increase the risk of statin-induced myopathy in women (120). Women may also show different responses to drugs as seen in renin-angiotensin-aldosterone system inhibitors. For example, the preference of angiotensin receptor-neprilysin inhibitors (ARNI) in women with heart failure and preserved ejection fraction as in PARAGON-HF trial showed positive effect on the endpoints in women whereas ARNI did not improve endpoints in men (121). Accordingly, we think that AI-driven pharmacokinetic modeling might help in optimizing dosing regimens, minimizing adverse drug reactions of pharmacotherapies in women. Additionally, sex-specific variations in natriuretic peptides are conflicting findings among studies (10), yet such variation may affect responsiveness to diuretics, necessitating individualized fluid management strategies for female heart failure patients. Hormonal fluctuations during menstruation, pregnancy, and menopause further influence drug metabolism (9), emphasizing the need for dynamic dosing strategies based on the female life cycle which calls for more patient-centred approach in managing women with CVD (Figure 2).

## Precision medicine in international cardiovascular guidelines

Precision medicine is modestly discussed in the international cardiology guidelines. The 2021 European Society of Cardiology (ESC) guidelines on CVD prevention, highlighted the complexity of CVD prevention approach and advocated for a shift towards more digitalized platforms for risk calculation (122), emphasizing the importance of tailored interventions at the individual level. In the 2023 ESC guidelines on diabetes and CVD management, the recommendations on cardiovascular risk stratification, screening, diagnosis, and treatment of CVD in patients with diabetes were provided with a focus on individualized approaches to reduce cardiovascular risk, incorporating evidence from large cardiovascular outcome trials to guide personalized management strategies (123). Genetic testing in hypertrophic obstructive cardiomyopathy, familial dyslipidemia, and Atrial fibrillation have been historically reported in the European and American guidelines recommendations to guide specialized patients’ management (124–126). Patient risk stratification in pregnant women using different scores were well implemented in the ESC guidelines in managing CVD in pregnancy (77). However, despite its crucial role in optimizing patient care, precision medicine remains underrepresented in the international cardiology guidelines. This emphasizes the need for personalized risk assessment and management, underscoring the shift toward individualized, evidence-based strategies in cardiovascular care and practice.

Several limitations should be acknowledged when interpreting the findings of this review. This work adopts a narrative approach rather than a systematic review or meta-analysis; consequently, study selection was not based on predefined quantitative criteria, and direct comparisons or pooled performance estimates of cardiovascular risk models could not be performed. In addition, the intent to provide a broad overview of cardiovascular risk prediction in women; encompassing conventional risk scores, biomarkers, imaging modalities, omics-based approaches, artificial intelligence, and pharmacogenomics; inevitably constrained the level of critical detail achievable within each individual topic area.

Moreover, the evidence base informing many of the discussed tools and predictors is largely derived from high-income countries, which may limit the applicability of these findings to low- and middle-income regions. While emerging registry initiatives, such as the pilot Iraqi Registry of Cardiovascular Diseases in Women (IROCW) and the Middle East African Women CardioVascular Disease (MEA-WCVD) Registry (35, 127) represent important steps toward addressing this imbalance, the data generated to date remain early and geographically restricted. Finally, some commonly used clinical risk scores, including the HEART score, were not discussed in depth, as the focus of this review was on long-term cardiovascular risk prediction rather than short-term risk stratification in acute care settings.

**Figure 3:**
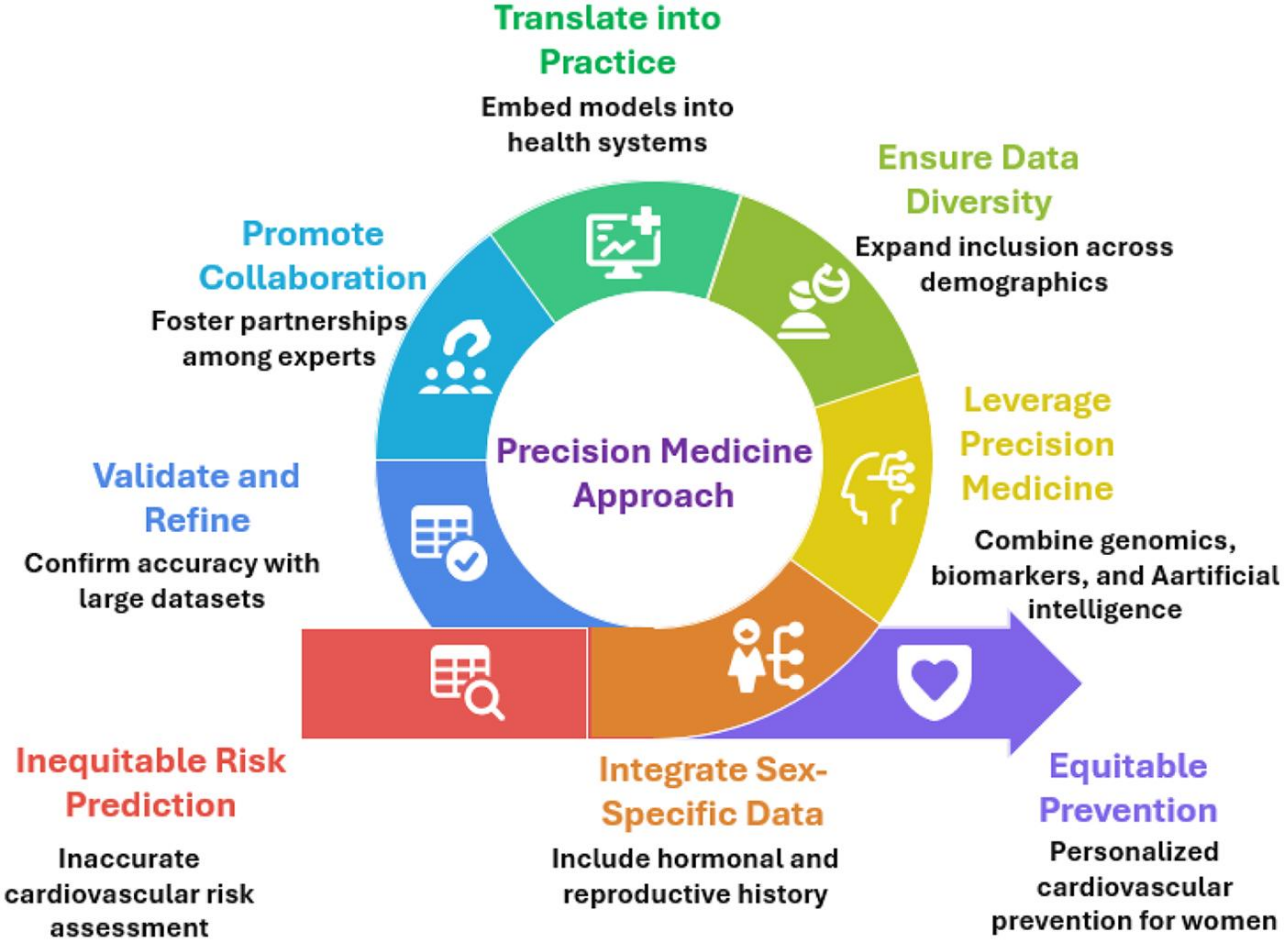
Future directions for advancing precision cardiovascular risk prediction in women.

Despite these limitations, emerging frameworks continue to guide the translation of precision medicine into cardiovascular care for women. Looking ahead, key priorities include expanding female representation in precision medicine research, validating sex-specific prediction tools, and ensuring equitable access to personalized interventions. Addressing these gaps will be crucial to translating precision cardiology into meaningful improvements in women's cardiovascular outcomes, see Figure 3.

## Conclusion

Improving cardiovascular risk prediction in women requires a comprehensive approach that considers biological, reproductive, and social determinants across the life course. Advances in precision medicine, such as genomics, biomarkers, imaging, and artificial intelligence, offer valuable tools to personalize assessment and guide prevention. Ensuring diverse representation in research and fostering collaboration among clinicians, scientists, and policymakers will be essential to translate these innovations into equitable and effective care for women worldwide.
